# Wandering Spleen in a Pediatric Lung Transplant Patient With Filamin A Deficiency: An Incidental Finding

**DOI:** 10.1111/petr.70161

**Published:** 2025-08-13

**Authors:** Savannah Ellis Knight, Mayel Yepez Donado, Maria Carolina Gazzaneo

**Affiliations:** ^1^ Division of Pediatric Pulmonary Baylor College of Medicine/Texas Children's Hospital Houston Texas USA; ^2^ Division of Pediatric Pulmonary and Critical Care Baylor College of Medicine/Texas Children's Hospital Houston Texas USA

**Keywords:** filamin A, pediatric lung transplant, wandering spleen

## Abstract

**Background:**

Filamin A (FLNA) deficiency is a known cause of progressive lung disease and need for pediatric lung transplant; however, what may be less well known to lung transplant providers are the extrapulmonary complications of FLNA deficiencies, such as wandering spleen. We present a patient who underwent a lung transplant for FLNA deficiency and later developed posttransplant abdominal pain.

**Case Presentation:**

An 11‐year‐old female who had previously undergone a bilateral lung transplant due to FLNA deficiency, causing progressive lung disease, presented with abdominal pain and diarrhea. The patient's stool was tested for causes of gastroenteritis using a gastrointestinal pathogen panel (GIPP). Additionally, an initial abdominal ultrasound was obtained to rule out surgical causes of acute abdomen. The initial abdominal ultrasound showed the spleen in the correct anatomical location. However, subsequent abdominal ultrasounds revealed an incidental finding of wandering spleen in multiple locations in the abdomen. As she has remained stable despite the migration of her spleen, the decision was made not to pursue surgical intervention and to continue monitoring with medical and surgical follow‐up.

**Conclusions:**

There are multiple complications caused by FLNA deficiency besides progressive respiratory failure, which include gastrointestinal (GI) complications such as wandering spleen. Wandering spleen is a rare clinical entity and, to our knowledge, this is the first case report of it being identified in a pediatric lung transplant patient. This case highlights the importance of transplant providers remaining vigilant when evaluating seemingly benign complaints such as abdominal pain in this population.

**Abbreviations**
CT: computed tomography
FLNA: filamin A
GIPP: gastrointestinal pathogen panel
LLQ: left lower quadrant
MRI: magnetic resonance imaging
PFT: pulmonary function tests
RLQ: right lower quadrant

## 1 Introduction

Wandering spleen is a rare condition characterized by abnormal mobility of the spleen due to pathological laxity, length, or absence of its ligamentous connections. This condition can be congenital or acquired, and the primary clinical concern is that torsion of the vasculature can occur, which can lead to acute splenic infarction [1]. Acquired causes of wandering spleen may be injury to the ligaments because of trauma, conditions that cause the ligaments to be more lax such as is seen in women of child‐bearing age, or in those with conditions such as filamin A (FLNA) deficiency [2, 3]. FLNA is an actin‐binding protein that regulates cytoskeleton reorganization, and it is essential for cell motility, migration, adhesion, response to stimuli, and structural integrity. Therefore, it is essential to the proper development of multiple organ systems. Loss of function of the FLNA gene, which encodes this protein, can cause abnormal neuronal migration, vascular and connective tissue anomalies, and is a known cause of interstitial lung disease [2]. Here we present a patient with FLNA deficiency with a history of lung transplant due to progressive respiratory failure who presents with abdominal pain and incidental finding of a wandering spleen.

## 2 Case Description

This is an 11‐year‐old female who first exhibited symptoms concerning for lung disease at birth. She required NICU admission for supplemental oxygen and was ultimately diagnosed with transient tachypnea of the newborn (TTN). She was able to wean to room air prior to discharge. However, she continued to experience persistent tachypnea. At 2 months of age, she presented to the pulmonary clinic for evaluation of chronic cough and ongoing respiratory symptoms such as increased work of breathing and tachypnea. Despite initial outpatient evaluation and treatment, she continued to worsen, requiring admission to the hospital with intubation and mechanical ventilation for respiratory failure. The workup for acute on chronic respiratory failure included chest computed tomography (CT) which showed mild peripheral pulmonary vascular attenuation, multilobar hyperinflation with hyperlucent lung parenchyma as well as scattered subpleural parenchymal opacities in the posterior upper lobes and bilateral lower lobes without honeycombing or septal line thickening to suggest fibrosis. Given these findings, FLNA deficiency was suspected. Magnetic resonance imaging (MRI) of the brain was obtained and showed periventricular heterotopia, further supporting the diagnosis. This was later confirmed with genetic sequencing showing the frameshift variant c.6584dupA, and she was ultimately diagnosed with X‐linked periventricular nodular heterotopia (X‐linked PNH). Although she was able to be extubated from the initial admission, her course was significant for rapid progression of respiratory failure, for which she was evaluated for lung transplant. Her transplant evaluation was routine, and as there were no gastrointestinal concerns, she did not receive abdominal imaging or other gastrointestinal‐specific tests. She was approved for transplant and received a bilateral lung allograft before one year of age.

**FIGURE 1:**
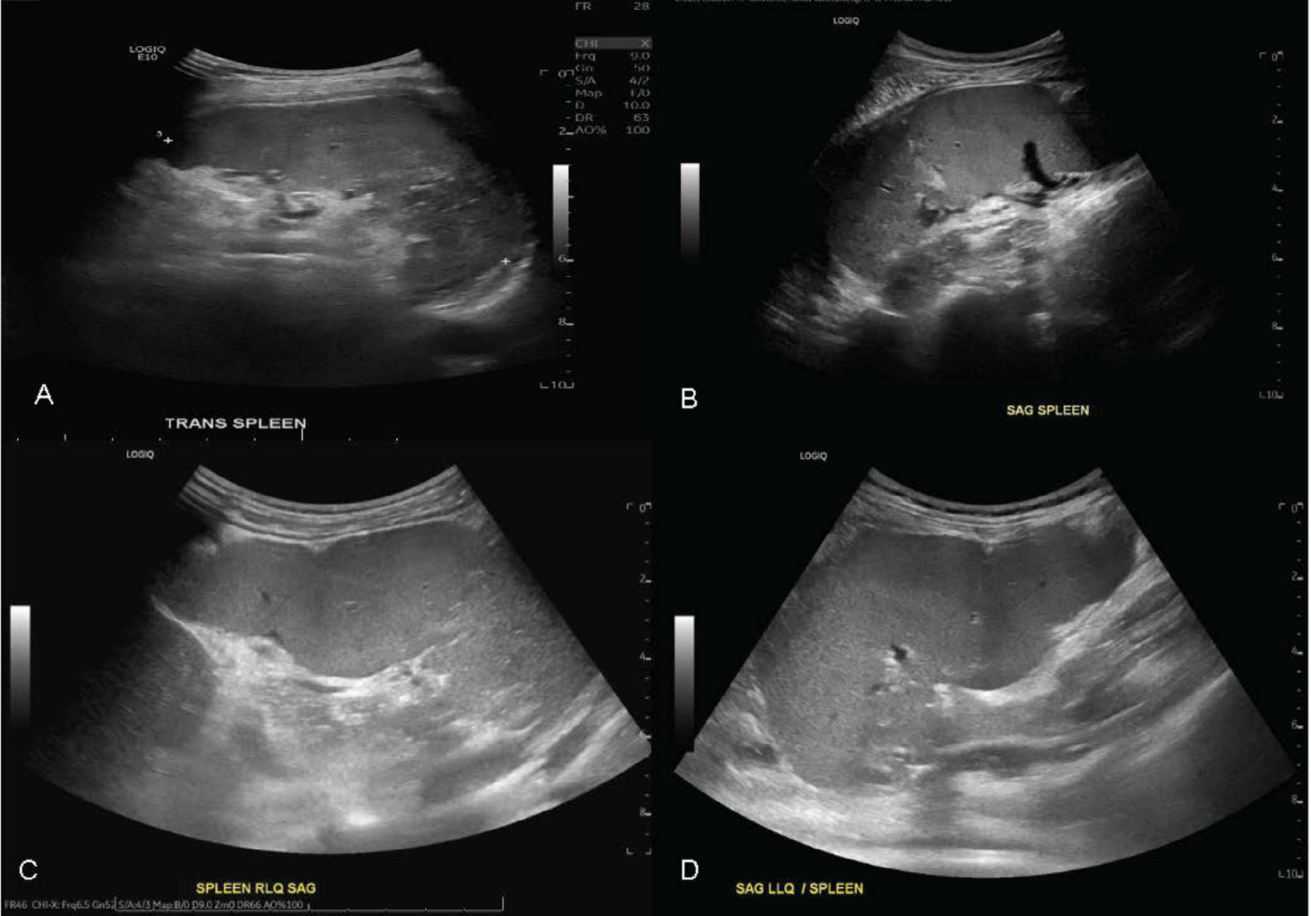
(A) Abdominal ultrasound showing heterogeneous echotexture in the inferior portion of the spleen without internal blood flow measured by Doppler (not shown), consistent with splenic infarct. (B) Abdominal ultrasound which shows normal spleen in correct anatomical positioning. (C) Abdominal ultrasound shows the spleen as “wandering” and located in the RLQ. Splenic blood flow measured by Doppler (not shown) was intact; though there was evidence of decreased flow velocity or vascular congestion. (D) Abdominal ultrasound showing wandering spleen now located in the LLQ. The ultrasound again demonstrated intact blood flow via Doppler (not shown) though vascular congestion.

She initially had an uneventful posttransplant course with good allograft function as evidenced by pulmonary function tests, until 8 years post lung transplant when the patient presented to the hospital with new diffuse abdominal pain, diarrhea, and fever. Abdominal ultrasound was concerning for infarction of the spleen; though otherwise had normal anatomical findings (Figure 1A). No etiology for infarction was found at that time, and she had no further abdominal complications or abdominal pain. Two years later, during which time allograft function began to decline and her chest imaging became concerning for bronchiolitis obliterans, she presented again with abdominal pain and diarrhea. An abdominal ultrasound was done on admission, showing normal size and location of all organs and was significant only for a small amount of free fluid in the abdomen and pelvis (Figure 1B). A gastrointestinal pathogen panel was completed, which showed evidence of sapovirus, and she was found to have a urine culture positive for enterococcus. Both were determined to be the cause of her abdominal pain at that time. With treatment, her abdominal pain improved. She then had recurrence of abdominal pain during the same hospitalization, now with abdominal distension, for which another abdominal ultrasound was performed. This ultrasound revealed an enlarged and “wandering spleen” which was errantly located in the right lower quadrant (RLQ) (Figure 1C). Doppler showed intact perfusion of the spleen; though there was evidence of vascular congestion suggestive of mild torsion. Pediatric surgery was consulted, and as her abdominal pain improved without intervention and blood flow to the spleen was intact, the team and family chose to monitor clinically with outpatient follow‐up and elective surgical removal.

She was readmitted one month later with symptoms of vomiting, diarrhea, and abdominal pain. Abdominal ultrasound now showed the wandering spleen located in the left lower quadrant (LLQ) with similar splenic vascular congestion but intact blood flow (Figure 1D). Gastrointestinal pathogen panel (GIPP) was now positive for norovirus, and she improved with symptomatic care, reassuring against the cause of her abdominal pain being from the wandering spleen. After this hospitalization, she established care with pediatric surgery outpatient to more closely monitor her symptoms and discuss the risks and benefits of surgical versus conservative management options. The surgical options discussed were splenectomy or splenopexy. As she is on chronic steroids and immunosuppression, she would be at higher risk of poor wound healing and postoperative infections. She also had a decline in lung function suspicious for bronchiolitis obliterans and rejection, so she would be considered higher risk to intubate for an elective procedure. In addition, splenic function seemed to remain intact as her recurrent gastrointestinal infections were attributed more to her immunosuppressive medication, and she had not had infections with encapsulated organisms. Since she has minimal symptoms of abdominal pain without nausea or vomiting, the decision was made to continue monitoring closely with a plan to intervene on the spleen if she started to have frequent pain attributed to this condition, or if there was ever evidence of torsion compromising blood flow to the spleen.

## 3 Discussion

Variants in FLNA can cause several X‐linked disorders depending on whether they result in loss or gain of function. Briefly, gain‐of‐function variants can cause the otopalatodigital spectrum disorders (OSD), which result in skeletal dysplasia with multi‐organ involvement [4]. Males with OSDs always have the disease, while females tend to have milder, if any, symptoms. Another disorder caused by FLNA gain of function is X‐linked cardiac myxomatous polyvalvular dystrophy, in which males show disease while females are asymptomatic [5]. Similarly, FLNA loss of function variants can cause different phenotypes depending on the genotype and whether the patient is male or female. Loss of function of FLNA can cause the development of the syndrome X‐linked PNH in both males and females [6]. PNH is characterized by neurons that failed to migrate to their appropriate location. This causes many nodules made of gray matter to line the ventricles of the brain, resulting in epilepsy, cognitive delay, mild learning disorders, and psychiatric conditions [4]. X‐linked PNH resulting from variants caused by a truncating mutation, typically a nonsense or frameshift mutation, may have a more severe phenotype with earlier presentation of symptoms such as in infancy or early childhood. Variants such as those from missense mutations may result in a somewhat functional protein, and symptoms may present later in adolescence or even adulthood [6]. However, females who are heterozygous for variants that cause a truncating mutation or whole gene deletion resulting in complete loss of function will have the syndrome, while hemizygous males with a complete loss of function variant are likely to suffer from early embryonic lethality or death in infancy [5]. Usually, only variants that lead to an incomplete or partial loss of function, such as missense mutations, distal truncating mutations, or mosaicism, result in the survival of males past infancy [7]. Both males and females may suffer from neurological sequelae of the disease alone or have multi‐organ involvement [5].

FLNA loss of function variants can lead to severe pulmonary manifestations and are a well‐known cause of childhood interstitial lung disease and a reason for pediatric lung transplant. In our institution, there have been seven transplants due to FLNA variants since 2002, all of which were also diagnosed with X‐linked PNH. The interstitial lung disease is due to a disorder of alveolar growth causing alveolar simplification, emphysematous lesions, and pulmonary vasculature pathology, often resulting in pulmonary hypertension [8]. The severity of this disease is such that lung transplant is unavoidable, and the average age at transplant is 11 months [9]. While interstitial lung disease caused by complete loss of function usually presents in infancy, variants causing incomplete deficiency have been reported to cause emphysematous lung disease that did not present until adulthood [10].

As was demonstrated in our patient, those with FLNA variants can develop gastrointestinal abnormalities, which can include wandering spleen [11]. Wandering spleen occurs because of abnormal peritoneal attachment, resulting in splenic ectopia due to the relaxation of ligaments in the spleen, kidney, and stomach [1]. The spleen can then migrate to various positions in the abdomen or pelvis, which can cause complex clinical situations. These clinical manifestations are variable, ranging from asymptomatic accidental discovery to acute abdomen caused by torsion of the vascular pedicle and splenic infarction. We now know that our patient, who had a history of splenic infarct of unknown etiology, likely had this complication because of laxity of peritoneal ligaments from FLNA deficiency, which caused torsion of her splenic vessels. Recent surgical literature regarding management of wandering spleen has favored preservation with splenopexy as opposed to splenectomy if the organ is viable. This is now the preferred surgical management to preserve the immune function of the spleen and avoid the common complication of postsplenectomy sepsis. Unfortunately, undergoing a splenopexy is not without risk. There is a high rate of reoperation or ultimately splenectomy due to splenic ischemia during surgery or recurrent symptoms of vascular torsion [12, 13]. In our patient, surgery was high risk due to her declining lung function, immunosuppression, and risk of poor wound healing from chronic steroid use; however, a splenopexy is likely to be preferred to preserve the immune function if surgery is unavoidable. Wandering spleen is not the only gastrointestinal complication that has been reported in patients with variants in the FLNA gene. X‐linked congenital chronic intestinal pseudo‐obstruction is a disease caused by FLNA deficiency and presents as mild intestinal hypomobility in females while it causes severe intestinal dysmotility in males [5]. Outside of this disorder, other gastrointestinal issues have been described, such as in the case series by Oegema et al. [7]; males with abnormalities in the FLNA gene suffered from various gastrointestinal problems including constipation, pyloric stenosis, congenital short bowel, intestinal pseudo‐obstruction, and malrotation. Similarly, in a case series of 47 male and female patients with various FLNA loss of function variants, multiple patients suffered from gastrointestinal complications, including a 15‐month‐old child who had a complete absence of gastrophrenic ligaments resulting in wandering spleen and another with torsion and volvulus of the stomach requiring extensive abdominal surgery. An additional patient in this series had severe constipation from pseudo‐obstruction requiring cecal flushes and eventually an ileostomy to relieve absent bowel movements [6].

Additional organ system involvement in loss of function variants includes the hematological, musculoskeletal, and cardiovascular systems. FLNA has a vital role in tethering platelets to blood vessels, so another complication seen in this disease is thrombocytopenia with or without abnormally large platelets, causing bleeding and poor wound healing [14]. Musculoskeletal abnormalities that have been reported include ligament and skin laxity causing joint hypermobility and skin fragility. Cardiovascular pathology includes congenital heart disease such as patent ductus arteriosus, atrial septal defects, abnormal cardiac valves, and aortic aneurysms [5]. Various miscellaneous abnormalities have been reported as well, including tracheobronchomalacia, dysmorphic facial features, irregular toe implant, and urogenital abnormalities [7, 8].

FLNA variants can cause a wide spectrum of abnormalities in many organ systems; though the presence and clinical implications of these abnormalities can vary greatly among patients. Lung transplant providers should be aware of the multisystem involvement and possible complications of FLNA deficiency, as they make up a fair number of our patients. Although this complication is rare and does not warrant routine screening, lung transplant clinicians should maintain a higher index of suspicion for serious conditions such as wandering spleen when these patients present with common and seemingly benign symptoms like abdominal pain. In such cases, a lower threshold for imaging may be warranted to rule out this potentially life‐threatening complication.

## Data Availability

Data sharing not applicable to this article as no datasets were generated or analysed during the current study.
